# Diesel soot aging in urban plumes within hours under cold dark and humid conditions

**DOI:** 10.1038/s41598-017-12433-0

**Published:** 2017-09-28

**Authors:** A. C. Eriksson, C. Wittbom, P. Roldin, M. Sporre, E. Öström, P. Nilsson, J. Martinsson, J. Rissler, E. Z. Nordin, B. Svenningsson, J. Pagels, E. Swietlicki

**Affiliations:** 1 Division of Nuclear Physics, Lund University, Box 118, SE-22100 Lund, Sweden; 2 Ergonomics and Aerosol Technology, Lund University, Box 118, SE-22100 Lund, Sweden; 3 Department of Physics, University of Helsinki, P.O. Box 64, 00014 Helsinki, Finland; 4 Department of Geosciences, University of Oslo, Postboks 1022, Blindern, 0315 Oslo, Norway; 5 Centre for Environmental and Climate Research, Lund University, Box 118, SE-22100 Lund, Sweden

## Abstract

Fresh and aged diesel soot particles have different impacts on climate and human health. While fresh diesel soot particles are highly aspherical and non-hygroscopic, aged particles are spherical and hygroscopic. Aging and its effect on water uptake also controls the dispersion of diesel soot in the atmosphere. Understanding the timescales on which diesel soot ages in the atmosphere is thus important, yet knowledge thereof is lacking. We show that under cold, dark and humid conditions the atmospheric transformation from fresh to aged soot occurs on a timescale of less than five hours. Under dry conditions in the laboratory, diesel soot transformation is much less efficient. While photochemistry drives soot aging, our data show it is not always a limiting factor. Field observations together with aerosol process model simulations show that the rapid ambient diesel soot aging in urban plumes is caused by coupled ammonium nitrate formation and water uptake.

## Introduction

Diesel exhaust particles (DEP) have large but poorly quantified negative impacts on both the Earth’s climate and human health. DEP contribute a significant amount of black carbon (BC) to the atmosphere. BC is warming the climate by absorbing sunlight and has been estimated to be the second largest contributor to anthropogenic radiative forcing^1,2^. (In this research, “DEP” refers exclusively to black carbon containing particles. Primary particles without BC content, which are also emitted from diesel engines, are not included in DEP.) DEP also cause a range of adverse health effects, and diesel exhaust is classified as carcinogenic^3,4^. While fresh diesel soot particles consist mostly of BC and are aspherical and non-hygroscopic, aged particles have lower BC fractions and are spherical and hygroscopic. Hence, the optical properties of DEP are altered upon atmospheric processing, due to absorption amplification^5–7^ upon condensation of secondary material (lensing) and restructuring of the soot core^7–9^. In addition to light absorption, DEP have other impacts on the climate system, related to aerosol dynamics and cloud microphysics. The efficiency of DEP as nuclei for cloud droplets^10^ – and possibly also ice nuclei^11^ – is expected to evolve during atmospheric processing. Furthermore, because wet scavenging is the main removal mechanism, the aging timescale determines the atmospheric lifetime, dispersion and vertical distribution of DEP^10,12^. In addition, effects on human health are likely altered as changes in particle size, shape and water uptake influence the deposition probability and site in the respiratory tract^13,14^.

However, quantifying the various consequences of DEP due to inhalation, light absorption, and interactions with water vapor, remains an active area of investigation. These investigations are hampered by the inherent properties of DEP. Their agglomerated, highly non-spherical shapes complicate particle sizing, and the chemical composition is difficult to measure because the main component – black carbon – is not amendable to conventional analytic chemistry techniques. In addition, aged DEP consist in part of semi-volatile material, which makes collection for *ex-situ* analysis prone to sampling artifacts.

A well-established technique that quantitatively measures the chemical composition of non-refractory particulate matter below 1 µm in diameter (nR-PM_1_) is electron ionization Aerosol Mass Spectrometry^15–17^ (AMS). Previous AMS deployments have identified a fraction of organic particulate matter, HOA (hydrocarbon-like organic aerosol), which is isolated through a source apportionment model and closely resembles the semi-volatile organic fraction of fresh DEP. In addition, this fraction is similar to the lubrication oil used in diesel engines^18–20^. However, as the traditional AMS technique is limited to non-refractory material, the main component (soot core) of DEP is not measured. Recent developments of the instrument have resolved this problem by the addition of a Soot Particle module, SP-AMS^21^. The SP-AMS utilizes an intra-cavity laser (λ = 1064 nm) which heats absorbing components such as refractory black carbon (rBC) until sublimation, thus extending the technique to include these components. The usefulness of the SP-AMS has been demonstrated in laboratory^10,22^ and field^23–26^ experiments involving DEP.

The aim of this study is to elucidate the processes involved in the atmospheric transformation of diesel soot, and to determine the timescales on which they operate. We report results from three sets of experiments: (i) urban (roadside) ambient sampling impacted by fresh DEP; (ii) rural, ambient sampling impacted by aged DEP; and (iii) simulated atmospheric aging of DEP using a smog chamber. Complementary *in-situ* techniques were used to measure the relationship between DEP size and mass (Differential Mobility Analyzer-Aerosol Particle Mass analyzer^27^, DMA-APM) and interactions with sub-saturated (Hygroscopic Tandem Differential Mobility Analyzer^28^, H-TDMA) and supersaturated (cloud condensation nuclei counter^29^, CCNC) water vapor.

Earlier studies have investigated the simulated aging of DEP proxies – flame generated soot^9,30,31^ – as well as true DEP^32^. There are also previous ambient *in-situ* observations of the microphysical properties of fresh and aged DEP, and the transition between them^9,23,33^. In this study, we compare experimentally simulated and ambient DEP processing. The ambient conditions, representative of mid-latitude winters, are markedly different from those typically reported in the literature. We show that diesel soot ages rapidly under cold, dark and humid conditions through interactions with particulate nitrate and water.

## Results and Discussion

### Time series analysis confirms urban plume influence on rural air

**Figure 1:**
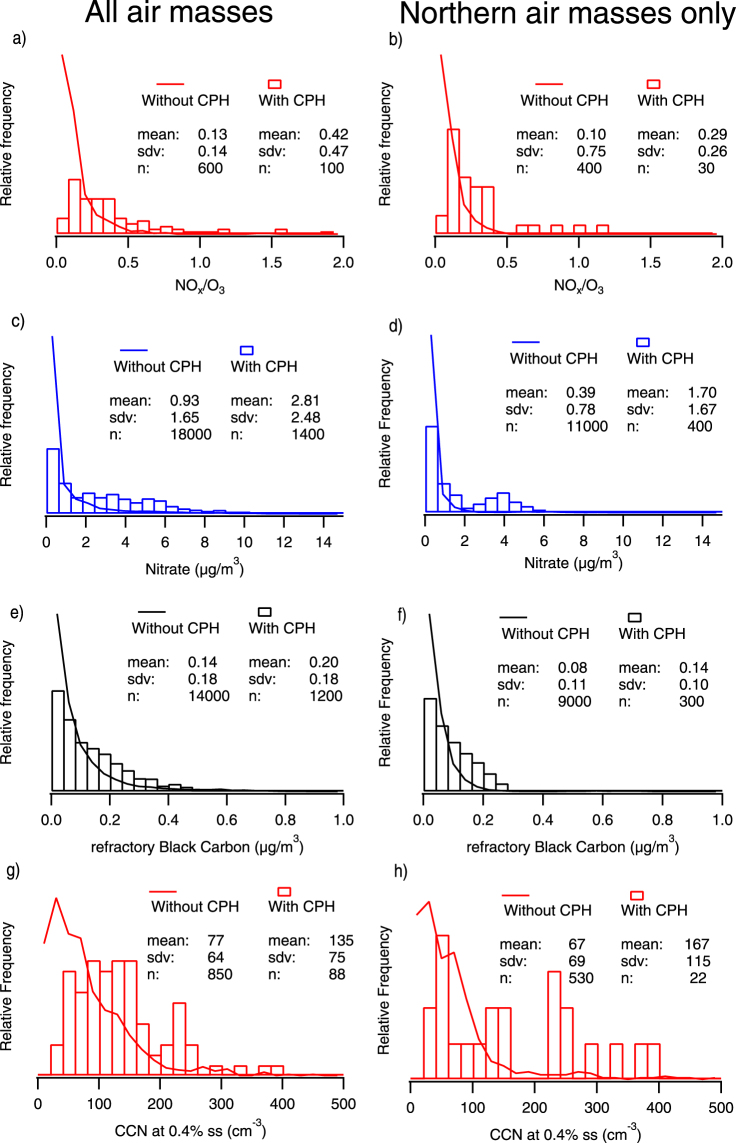
Normalized histograms for rural air passing (bars) and not passing (lines) Copenhagen upwind. Left: full campaign, right: northern air masses only. (**a**,**b**) NO_x_/O_3_, hourly data. (**c**,**d**) Particulate nitrate, 1 minute data. (**e**,**f**) Refractory black carbon (rBC), 1 minute data. g, h: cloud condensation nuclei (CCN) at 0.4% supersaturation (ss), hourly data.

The back-trajectory analysis showed that during approximately 10% of the rural campaign at Vavihill, Sweden^34^, the air sampled had passed the Copenhagen metropolitan area a few hours upwind. In order to facilitate our investigation of the urban plumes, we combined data from these periods, henceforth referred to as the “urban plume influence” subset. Similarly, we created a subset from our urban data, in which the fresh traffic emissions are enhanced; termed the “fresh traffic dominated” subset. Both subsets are further explained below. Air that had passed Copenhagen before arriving at the rural station was influenced by recent urban emissions, as can be seen in Fig. 1. This influence was clearly manifested in the ratio of NO_x_ (principally from traffic) to ozone (depleted in the urban plume) and in the concentration of particulate nitrate, which was on average four times higher in the plumes (2.8 µg/m^3^ vs 0.7 µg/m^3^ NO_3_). A recent study has shown a significant organic contribution to particulate nitrate in Europe^35^, yet >85% of the nitrate detected here was inorganic, both with and without plumes. Higher abundances of refractory black carbon (rBC) were also observed in the plumes from Copenhagen, 200 ng/m^3^ vs 140 ng/m^3^. The measured rBC mass concentration during the urban plume influence at Vavihill was also well correlated with the NO_x_ concentration with a linear 0.5-quantile regression slope of 0.0276 (rBC μg m^−3^)/(NO_x_ ppb_v_) (Supporting Information, Figure S1). The values of the rBC to NO_x_ ratio observed in the plumes were in good agreement with the values recently found in high traffic flow areas^36^. Furthermore, in-plume cloud condensation nuclei (CCN) concentrations were elevated throughout the range of measured supersaturations (SS 0.1% to 1.4%, see Supporting Information, Figures S2 and S3). As expected, the number of extra CCN contributed by the plumes increased at higher SS, e.g., at 1.4% approximately twice the number concentration found at 0.4% was measured. The average total number of particles also increased in the plumes by a factor of ~2. More than 80% of the increase in number concentration was due to particles between 30 and 300 nm (mobility equivalent diameter, see Figure S4).

**Figure 2:**
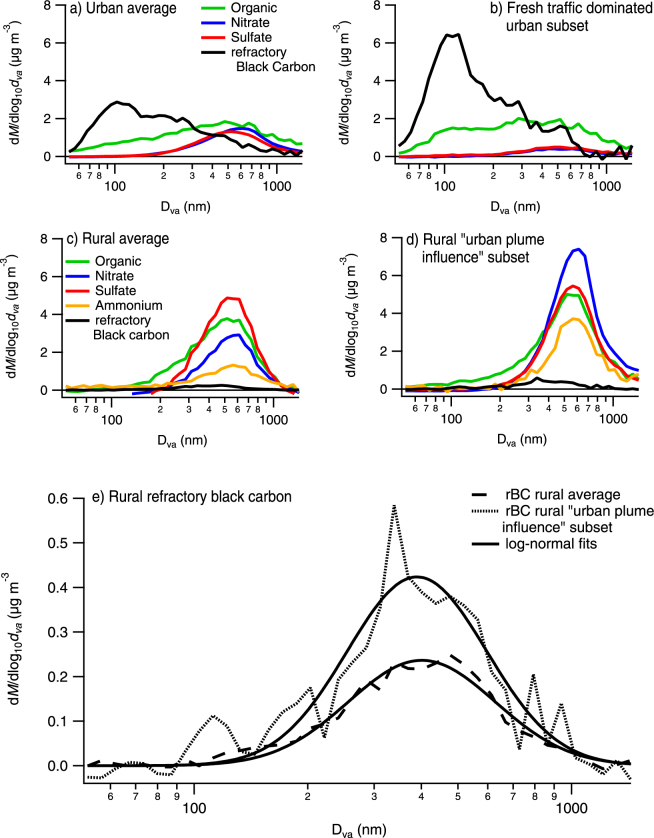
Chemically resolved mass weighted aerodynamic size distributions. Urban average (**a**), Fresh traffic dominated urban subset (**b**), rural average (**c**) and rural, urban plume influence subset (**c**). (**e**) comparison of the rural refractory black carbon distributions, with log-normal fits.

The air sampled was of northern origin more than half of the time (~60%). The northern air masses had less frequently passed Copenhagen for geographical reasons (see Supporting Information, Figure S7.) However, as air masses arriving from the north had a lower contribution from long-range transport, the urban influence was more pronounced for the northern air masses. Inspection of the northern air mass back-trajectories confirmed that mainly low-emission regions were passed before Copenhagen. The averaged result of the campaign is similar to that of the northern air masses (see Figs 2 and S5). This implies that the Copenhagen plume influence was not confounded by source region: the additional pollution originated from Copenhagen rather than continental Europe.

### Size-resolved chemical composition of fine particles in urban and rural ambient air

Black carbon was more abundant in the urban air compared with the rural air sampling, due to the proximity of large numbers of diesel-powered vehicles. However, in both urban and rural air, the majority of PM_1_ (the mass concentration of fine particles) was due to regional background: accumulation mode particles with a mass mode diameter around 600 nm (D_va_). To enhance the relative contribution of fresh diesel exhaust particles in the urban dataset, a fresh traffic dominated subset was defined based on the chemical composition of the particles. The data in which rBC accounted for more than half of the particulate mass were selected for the subset (14% of the data). This approach captures the minute-to-minute variability of fresh DEP influence in the dataset due, for example, to the variable dilution of the sampled traffic exhaust. For the rural sampling, the data were instead filtered based on back-trajectory analysis to create a subset with influence from Copenhagen’s plumes, as previously described and further explained in the method section.

The size (D_va_) distribution of rBC observed in the urban air features a mode around 100 nm, which is consistent with previous studies of fresh diesel exhaust particles^37^. On average there were very low fractions of nitrate and sulfate (less than 2%) below 200 nm (D_va_). The rural rBC distributions had no rBC mode around D_va_ 100 nm that would correspond to fresh emissions. This indicates that the DEP sampled at the rural site had grown to larger sizes during the few hours of transit (see Table 1). In both urban and rural air, a significant amount (~5% by mass) of rBC was present in the accumulation mode, most likely due mainly to long-range transport.

**Table 1:** Plume conditions during transit from Copenhagen to rural station, from HYSPLIT4.

	Transit time [h]	Relative Humidity [%]	Temperature [C]	Irradiance [W/m2]
Average (rBC weighted)	5	86	−1	59
Max	23	99	4	152
Min	2	74	−6	0

The rural rBC distribution shows a surprisingly small size shift in the urban plume influence subset: from 401 +/− 11 nm (95% CI) in the average distribution to 390 +/− 22 nm for the subset. However, as shown in Fig. 1, a significant fraction of rBC in the urban plume influence subset originated from the Copenhagen metropolitan area (~40% higher rBC concentration). Yet in Copenhagen, much smaller (D_va_~100 nm, see Fig. 2) rBC containing particles were observed as previously discussed. The average and the subset of rural rBC distributions are plotted isolated from the rest of PM_1_ in Fig. 2e. Fitted log-normal distributions are also shown.

We investigated the effect of differing source regions upwind of Copenhagen on the comparison of average and urban-plume-influenced rural data. Air masses of northern origin with low contributions from long-range transport were also compared depending on if the air had had passed Copenhagen or not. In this comparison, the rBC concentrations were about 80% higher in the air which had passed Copenhagen. The result again showed little effect on the size distribution: 370 +/− 25 nm and 395 +/− 97 nm for the average distribution and urban plumes, respectively (Figure S5), despite the large relative increase in rBC concentration.

Given that the additional rBC observed at the rural site did originate from Copenhagen and had similar properties upon emission as the fresh DEP observed at roadside, the rBC distributions imply extensive atmospheric transformation in the plumes during the transport from the Copenhagen metropolitan area to the regional background station only 60 km downwind. Thus rapid transformation from fresh to aged DEP occurred under cold, dark and humid conditions (listed in Table 1).

### Synthesis of field and laboratory diesel exhaust particle aging

Upon emission, diesel exhaust particles are highly aspherical agglomerates^38^ of black carbon spherules coated with lubrication oil. However, atmospheric processing induces extensive transformation of DEP size, shape and composition, which in turn alters the particles’ environmental impact (light absorption and scattering, water vapor uptake, and lung deposition). In our smog chamber experiments, freshly emitted DEP were coated with condensing secondary organic material^10,39,40^ and the particle mass fraction of rBC decreased gradually. As previously reported, the aging resulted in progressively increased hygroscopicity^10^.

The freshly emitted DEP observed in urban air contained high fractions of rBC and had small D_va_ (~100 nm), while the atmospherically processed rBC sampled at the rural site was heavily coated and of larger D_va_. The smog chamber data illustrate the transition from freshly emitted to atmospherically processed DEP. Condensation onto the particles caused the increase in D_va_ as they gained mass, and transformed from agglomerated shapes into spheres.

**Figure 3:**
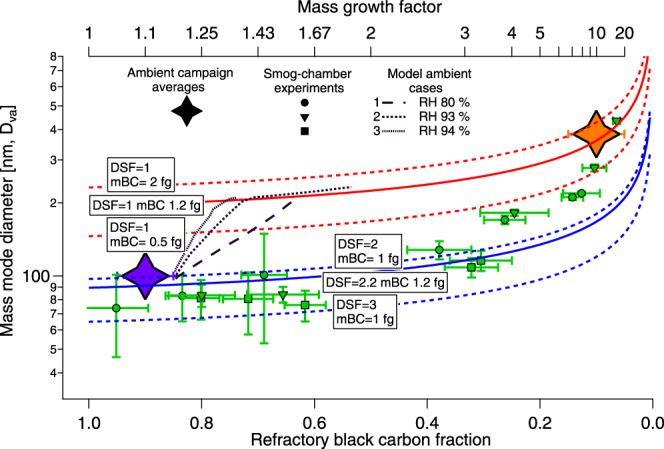
Mode particle diameter (D_va_) as a function of rBC mass fraction. Copenhagen (urban, blue star), Vavihill (rural, red star) and three diesel exhaust smog chamber experiments. The smog chamber error bars denote precision of log-normal fitted mode diameter (95% CI) and three standard deviations (n = 5–10) of the measured rBC fractions. For ambient data (stars), the rBC mass fractions were measured with Differential Mobility Analyzer-Thermal Denuder-Aerosol Particle Mass Analyzer (DMA-TD-APM), and the error bars give the range of the observed non-volatile fraction (Rissler *et al*.^48^) for each mode. Solid lines represent agglomerated (blue) and spherical (red) particles both containing 1.2 fg BC. Dashed lines illustrate the implications on particle mass and shape. Dashed black lines show model results for 1.2 fg BC particles for three model ambient cases of trajectories between Copenhagen and Vavihill, see Table 2. Details in text.

The effects of particle shape (dynamic shape factor [DSF]: dependent on drag force relative to spherical particles) and rBC mass on our data are explored in Fig. 3. The solid lines in the figure indicate the evolution of two hypothetical 1.2 fg BC particles with different morphologies: the red line shows a spherical particle, while the blue line represents a non-spherical (DSF 2.1) particle. The shape factor of the non-spherical particle, and the rBC mass of 1.2 fg, was chosen based on our combined SMPS and DMA-APM laboratory data on fresh DEP. Single particle incandescence measurements performed in traffic-dominated wintertime air in Paris 2012^41^ show a similar mass weighted mean value, 0.9 fg of refractory black carbon. The shape factor we used is consistent with laboratory investigations of fresh diesel exhaust particles^38^.

A fresh DEP is approximately 100 nm smaller in D_va_ compared with an equivalent spherical particle with the same mass and material density. Furthermore, a hypothetical DEP particle that retains its shape during aging is approximately 200 nm smaller in D_va_ at rBC fraction 0.1 compared with a (more realistic) spherical accumulation mode DEP. As shown in Fig. 3, the DEP found in the accumulation mode in the ambient data had a DSF near 1.0 and were thus spherical. In the smog chamber, the shape shift predominantly occurred at the later stages of the simulated aging, when the DEP were dominated by secondary organic material (rBC fraction around 0.05–0.3), which is consistent with recent laboratory investigations with flame soot and secondary organic material under dry conditions^31^. However, as the rBC mass added by the urban plumes was in the 200–400 nm D_va_ range (see Fig. 1), the particles where spherical earlier in the aging process (at higher rBC fraction) as illustrated by three model ambient cases (see Fig. 3) and further discussed below.

### Implications for diesel soot aging in urban plumes

Our field data show a rapid atmospheric transformation of diesel soot. This can be deduced from the similarities between rBC size distributions measured at the rural site with and without urban plume influence (Fig. 2e) and the high sensitivity of the sizing employed towards particle shape (see Fig. 3). While the ambient aging timescale was similar in our smog chamber experiments (approximately five hours), the photochemistry was significantly less intense in the field campaign. The OH exposures calculated using the Aerosol Dynamics, gas phase CHEMistry and radiative transfer Model (ADCHEM) differ by one to four orders of magnitude, and furthermore, the ratio of precursors of condensable material to DEP was higher in the laboratory. Consequently, atmospheric processing compacts the shape of diesel soot very rapidly in urban plumes under humid, low sunlight and low temperature conditions despite the limited photochemistry. For comparison, Peng *et al*.^9^ recently found similar timescales compared to our study for BC transformation under polluted conditions in Beijing, while slower rates were reported for the less polluted air (yet with respect to our study, more polluted) in Houston, Texas.

Liquid water, because of its high abundance and surface tension, was in all likelihood the cause of the rapid soot compaction found in the field in this study. Our smog chamber H-TDMA data for secondary organic material (SOA) dominated aging in the absence of ammonium nitrate (Supporting information Figure S6) shows that at elevated relative humidity (RH 90%), restructuring begins in the early stages of aging. Furthermore, the water induced morphological evolution towards spherical particles is completed faster than during dry conditions, caused by secondary organic material alone. However, no restructuring occurred upon elevated RH (90%) for fresh soot, as fresh DEP are non-hygroscopic^10,32^ which is also consistent with the low D_va_ of fresh rBC in urban environments. Thus, the SOA enabled water uptake by aging DEP^10^ in our smog chamber experiments.

During our ambient campaigns (RH 80–100%), the water induced transformation was likely enabled mainly by ammonium nitrate rather than SOA. Other species than inorganic nitrate may nevertheless also contribute to DEP ageing, including SOA which can form despite low photochemistry e.g. due to autoxidation^42^. As ammonium nitrate yields significantly more water soluble entities per condensed mass than SOA, less of it is required in order to partition the same amount of liquid water into aging DEP, as further discussed below. Our field observations are consistent with laboratory soot coating studies with the DMA-APM technique. It has been shown^43^ that soot coated with sulphuric acid and subjected to high RH cycling (RH = 90%) approaches spherical shapes already at a BC mass fraction of about 0.3–0.5. Our previously reported smog chamber aging of light-duty gasoline vehicle exhausts^39^, which are abundant in the Copenhagen plume, featured high ammonium nitrate formation. There are also numerous other anthropogenic activities in Copenhagen and en route that provide precursors. Specifically, ammonia is emitted in high abundance from agriculture, while traffic is a strong source of NO_x_. Furthermore, nitrate and ammonium are the chemical species that are most enhanced when filtering the rural data for Copenhagen influence (see Fig. 2), and the modeling of urban plume aging also supports extensive ammonium nitrate formation^44^.

Three representative cases were selected for detailed aerosol process simulations (see Supporting information Figure S7). As the fresh DEP constitute a substantial fraction of the condensation sink available for secondary PM formed in the plume, between 6 and 22% of the formed ammonium nitrate adds to secondary coatings of the DEP according to the ADCHEM simulations. This together with the co-condensing water produces a DEP mass growth factor >2 (Figures S12–S14). This results in nearly complete soot particle restructuring and a factor of ~2 increase in D_va_ between Copenhagen and Vavihill (model results in Fig. 3). The different slopes of the modeled D_va_ evolution for Cases 1, 2 and 3 in Fig. 3 are caused by the different RH conditions. At high RH (Cases 2 and 3), much less dry particle inorganic coating material is required to reach complete soot particle restructuring compared to less humid conditions (Case 1). Table 2 describes the plume conditions for the model simulations in Cases 1–3, the modeled cumulative OH exposure, NH_3_ concentration just upwind Copenhagen and the nitrate, BC, NO_x_ and O_3_ concentration changes induced by the Copenhagen urban plume at Vavihill.

**Table 2:** Plume conditions, modeled cumulative OH exposure, NH_3_ concentration, and nitrate (NO_3_), BC, NO_x_ and O_3_ enhancement/suppression at Vavihill due to the Copenhagen urban plume, for Cases 1–3.

Case	Transit time [h]	RH (%)	Temp [C]	Irrad [W/m^2^]	OH [molec/cm^3^ h]	NH_3_ (ppb_v_)	*ΔNO_3_ [μg/m^3^]	*ΔBC [μg/m^3^]	*ΔNO_x_ [ppb_v_]	*ΔO_3_ [ppb_v_]
1	5	80	−4	~100	3.8 × 10^5^	3.5	2.2	0.6	14	−10
2	2	93	+2	0	1.4 × 10^4^	6.5	2.2	0.2	4	−3
3	2	94	+2	~50	3.2 × 10^5^	8.1	1.2	0.2	12	−5

*Values derived by taking the difference between the model results inside (at Vavihill) and outside the urban plume from Copenhagen.

The ammonia concentration upwind Copenhagen is relatively high because of the extensive agricultural sector in Denmark and southern Sweden. Thus, according to the model simulations, the wintertime nitrate formation between Copenhagen and Vavihill is not limited by the amount of ammonia available to neutralize the inorganic particle phase. In the daytime, the secondary nitrate formation is instead limited by the rate at which HNO_3_ is formed from the reactions between OH and NO_2_ originating from the traffic in Copenhagen. During nighttime, the nitrate formation is instead governed by formation and heterogeneous conversion of N_2_O_5_^45–47^. Thus, despite the low wintertime solar irradiance and limited OH exposure, substantial nitrate formation always occurs within timescales of less than ~8 hours.

In addition to the condensation pathway we studied in the laboratory and have invoked to explain our ambient observations, there exists an alternative route: coagulation, through which DEP may evolve in the space defined by Fig. 3. By coagulating on a pre-existing accumulation mode particle, DEP will instantaneously shift to the accumulation mode. As this occurs, the resulting particles could have similar aerodynamic properties and chemical composition as the ones that grew into the accumulation mode through condensation. According to the ADCHEM simulations, 9–32% of the BC mass from the diesel vehicles in Copenhagen coagulates with the background accumulation mode during the transport (2–5 h) from Copenhagen to Vavihill. The coagulated rBC forms a separate BC particle mode with a dry particle geometric mean diameter (GMD_va_) between 250 and 460 nm. In the model, this mode is partly separated from the BC particles that are only coated due to condensation (dry particle GMD_va_ of between 145 and 160 nm) (Figure S15).

Our results show that the atmospheric transformation of diesel exhaust particles occurs rapidly, over a few hours, under the cold, damp and dark conditions that are typical of mid-latitude wintertime. While our data clearly show that the majority (by mass) of DEP are transformed, earlier investigations have shown that some DEP are only moderately aged during the transit from Copenhagen to Vavihill^48^ under similar conditions. These moderately transformed DEP were detected in fresher plumes (residence time 2–3 h, see Table 1 for comparison) and contributed approximately 20% of the total particle number concentration.

The increased concentration of cloud condensation nuclei observed in the urban plumes is consistent with the ammonium nitrate coating the aging diesel exhaust particles. The gradual increase of added CCN with supersaturation (SS, see Figures S5 and S6) reflects the distribution of CCN potential in the plumes. At SS 0.4%, half of the potentially active (as defined by the measured concentration at SS 1.4%) particles act as CCN. This is the expected critical supersaturation of a spherical 65 nm DEP with an rBC mass fraction of 0.4 if coated with ammonium nitrate, or an rBC mass fraction of 0.1 if coated by SOA.

Our model descriptions illustrate that the ambient conditions, particularly the relative humidity, influence the rate of the aging process. The rapid DEP transformation was largely caused by inorganic chemistry involving anthropogenic nitrogen emissions, notably ammonia from agriculture and NO_x_ from traffic, rather than the more complex organic chemistry which occurred in the plumes. This is because ammonium nitrate formation is generally faster than SOA formation from anthropogenic volatile organic compounds (VOCs)^44^, and NO_x_ is co-emitted with DEP in high abundance. Furthermore, inorganic coatings age DEP more efficiently than organic coatings, both in terms of water uptake and, as a consequence, restructuring. Thus, from the perspective of diesel soot aging in urban wintertime plumes, understanding the formation of the inorganic secondary particulate mass may be more important than understanding the SOA formation.

## Methods

### Trajectory analysis

The trajectory model HYSPLIT4^49^ was used to determine if the sampled air masses had passed the Copenhagen metropolitan area, bounded by a 21 × 25 km rectangle, on their way to the Vavihill rural station. The trajectories were also used to determine the air mass origin by calculating the azimuth angle between Vavihill and the center of gravity of 5-day backward trajectories. The trajectories arrived at 100 m above ground level and were calculated hourly. The meteorological data used to calculate the trajectories were provided by the NCEP (National Centers for Environmental Prediction) Global Data Assimilation System (GDAS).

### ADCHEM modeling of diesel soot aging in the urban plume

In order to estimate the typical diesel soot aging in the urban plume from Copenhagen, three representative HYSPLIT trajectories (Cases) were chosen for model simulation with the 2-dimensional version of the Lagrangian Aerosol Dynamics, gas phase CHEMistry and radiative transfer model ADCHEM^50,51^ (See Figures S7 and S8 in the Supporting Information). To be able to follow the gradual transformation (coating mass fraction, hygroscopic growth, coagulation and compaction) of the freshly emitted diesel soot particles in Copenhagen, they were represented with a separate externally mixed particle number size distribution only for soot particles emitted in Copenhagen. Additionally, the ADCHEM model tracked the fraction of the soot particles from Copenhagen that coagulated with the long-distance transported background particles in and downwind Copenhagen. This was achieved by representing these particles with a third particle number size distribution that only comprised the soot particles from Copenhagen that had coagulated with long-distance transported background particles. ADCHEM was also equipped with a new scheme for soot particle restructuring based on experimental results on soot particles coated with sulfuric acid and water^43^. This scheme represented the freshly emitted DEP as fluffy agglomerates with a size dependent effective density parameterization based on experimental data from the same measurement campaign (Rissler *et al*.^48^). When the particles are coated with inorganics (water, ammonia, nitric acid, sulfuric acid, etc.) they gradually become more compact, which was manifested in increasing effective density, decreasing mobility diameter (D_m_) and increasing vacuum aerodynamic diameter (D_va_, further discussed below) of the soot particles as further described in Roldin *et al*. (in preparation).

The gas-phase chemistry in ADCHEM was simulated using the near-explicit Master Chemical Mechanism version 3.3.1^52–56^ (MCMv3.3.1). In total 25 non-methane volatile organic compounds (NMVOCs) (listed in the Supporting Information) were used to represent the anthropogenic VOCs. In total, the gas-phase mechanism comprised 3,509 species and 10,504 reactions. The emissions of NVOCs, NO_x_, SO_x_, CO and NH_3_ with 0.1° × 0.1° resolution were retrieved from the EMEP (European Monitoring and Evaluation Programme) database (EMEP/CEIP 2014, Present state of emissions as used in EMEP models: http://www.ceip.at/webdab_emepdatabase/emissions_emepmodels/). However, for the Copenhagen region and southern Sweden, we instead used the 1 × 1 km^2^ resolution emissions of CO, SO_2_, NO_x_ and NMVOCs from the Environmental Department, City of Malmö^57^. According to the model simulations, the daytime OH concentration in the urban plumes was relatively sensitive to the NMVOC emissions in Copenhagen. In order to get the modeled absolute nitrate mass concentration and estimated nitrate mass enhancement in the urban plume to agree with the AMS observations at Vavihill for Cases 1 and 3, we chose to multiply the NMVOC emissions by a factor of 5. The model results presented in this study are from simulations using this assumption. Without this assumption, the cumulative OH exposure and nitrate formation in the urban plume for Cases 1 and 3 was reduced by a factor of 2.

We ran ADCHEM with 40 horizontal and 20 vertical layers. The horizontal resolution was 1 km, and the vertical resolution 100 m. Thus, the model accounts for both vertical and horizontal dilution of the urban plumes^44,50^. The main model time step was set to one second. This short time step ensured that we were able to accurately simulate the non-equilibrium and particle size dependent condensation and dissolution of organic compounds, H_2_SO_4_, HNO_3_ and NH_3_, and the hygroscopic growth for both the externally mixed soot particles and the aged background particles.

### Ambient measurements

Ambient measurements were performed in Copenhagen, Denmark and at the rural background Vavihill station in southern Sweden. In Copenhagen, roadside air from a busy street was sampled, further described by Rissler *et al*.^48^ Approximately 27,000 vehicles per day passed the location (average value for 2012), ~35% of which were diesel powered light-duty vehicles. SP-AMS data were acquired from December 26, 2011 to January 22, 2012. The rural site Vavihill is part of the pan-European ACTRIS station network^58^ and is situated approximately 60 km northeast of the city of Copenhagen (see Supporting Information Figure S8), and further described by Kristensson *et al*.^34^. The rural SP-AMS dataset was acquired from January 10 to February 25, 2013.

### Smog chamber experiments

Smog chamber experiments were used to investigate the mechanisms through which diesel exhaust particles transform in the atmosphere. In the experiments, further described by Wittbom *et al*.^10^, DEP from an idling light-duty diesel powered Euro II vehicle were injected into a 6 m^3^ smog chamber spiked with light aromatic SOA precursors. The DEP were subjected to a cumulative OH exposure of approximately 8 * 10^6^ molecules/cm^3^ * h.

### Soot Particle Aerosol Mass Spectrometer

In the SP-AMS^21^ aerosol is sampled through an aerodynamic lens with near unity particle transmission in the range 100–600 nm^15^. The resulting particle beam is modulated to obtain particle time-of-flight resolved data, which is interpreted in terms of vacuum aerodynamic diameter (D_va_), a type of equivalent diameter^59^. This sizing, based on low-pressure drag force and particle inertia, is sensitive to particle morphology, a feature we exploited to investigate DEP aging. Post sizing, the particle beam is vaporized, and the vapors ionized by 70 eV electrons, to facilitate on-line time-of-flight mass spectrometry on the particle ensemble.

The means of vaporization determine which species are detected with the instrument. The SP-AMS is equipped with an intra-cavity laser (λ = 1064 nm) which heats absorbing components, predominantly refractory black carbon (rBC) in the experiments described in this research, until sublimation. In order to keep the capabilities of the (non-SP) AMS, we chose to retain the conventional tungsten vaporizer heated to 600 °C (i.e., we used two vaporizers). By switching off the laser, the (non-SP) AMS data were acquired.

### Data availability

The datasets generated during and/or analysed during the current study are available from the corresponding author on reasonable request.

## Electronic supplementary material

Supporting information for Diesel soot aging in urban plumes within hours under cold dark and humid conditions
