# Supplementary material for: Diesel soot aging in urban plumes within hours under cold dark and humid conditions
Source: Sci Rep. 2017 Sep 28;7:12364. doi: 10.1038/s41598-017-12433-0 (PMC5620063; doi:10.1038/s41598-017-12433-0)
Supplement: Supplementary file 1 — Supporting information for Diesel soot aging in urban plumes within hours under cold dark and humid conditions [file 41598_2017_12433_MOESM1_ESM.pdf]

Supporting information for

Diesel soot aging in urban plumes within hours under cold dark and humid conditions

A. C. Eriksson[1,2*], C. Wittbom[1], P. Roldin[1,3], M. Sporre[4], E. Öström[1,5], P. Nilsson[2], J. Martinsson[1,5], J. Rissler[2], E. Z. Nordin[2], B. Svenningsson[1], J. Pagels[2] and E. Swietlicki[1]

[1]Division of Nuclear Physics, Lund University, Box 118, SE-22100, Lund, Sweden

[2]Ergonomics and Aerosol Technology, Lund University, Box 118, SE-22100, Lund, Sweden

[3]Department of Physics, University of Helsinki, P.O. Box 64, 00014, Helsinki, Finland

[4]Department of Geosciences, University of Oslo, Postboks 1022, Blindern, 0315 Oslo, Norway

[5]Centre for Environmental and Climate Research, Lund University, Box 118, SE-22100, Lund, Sweden

[*] Corresponding author: Axel.Eriksson@design.lth.se

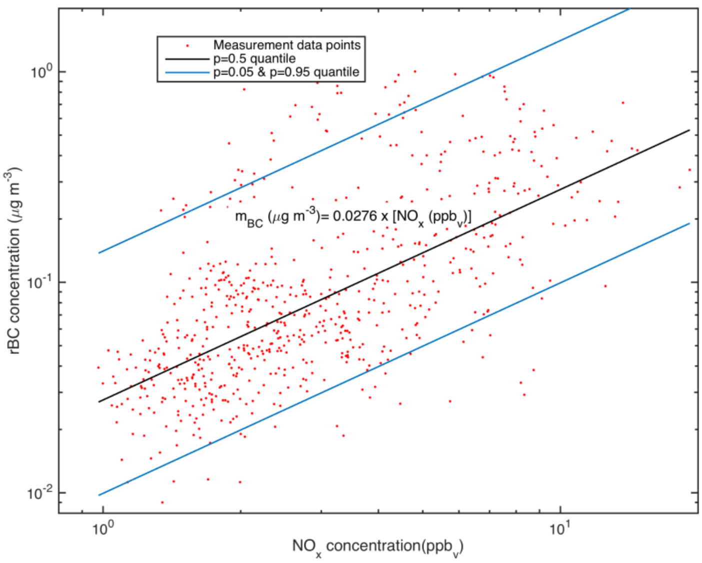

Figure S1. Scatter plot with the measured rBC mass concentration on the y-axis and the measured NOx concentration on the x-axis. The measurement points represent 1 hour average concentrations at Vavihill during time periods when the Copenhagen urban plume was estimated to influence the air quality at the station. Shown are also a fitted linear quantile regression fit (0.5 quantile), the 0.05 and 0.95 quantile.

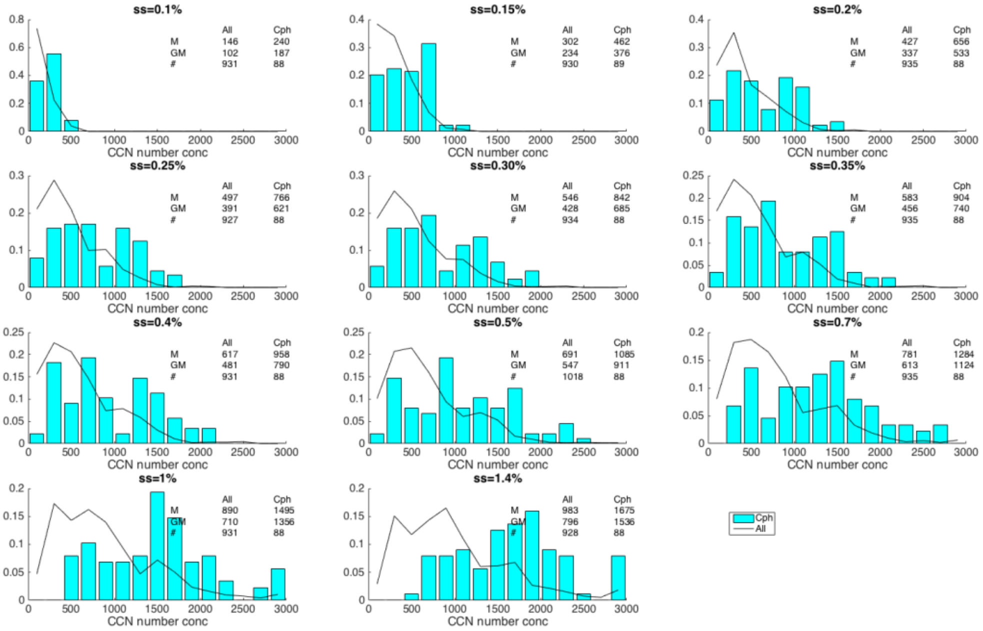

Figure S2. Histograms of cloud condensation nuclei (CCN) concentrations at the rural station with (bars) and without (lines) urban influence for the full campaign. Fraction of data vs concentration (cm^{-3}) at varied supersaturation (ss).

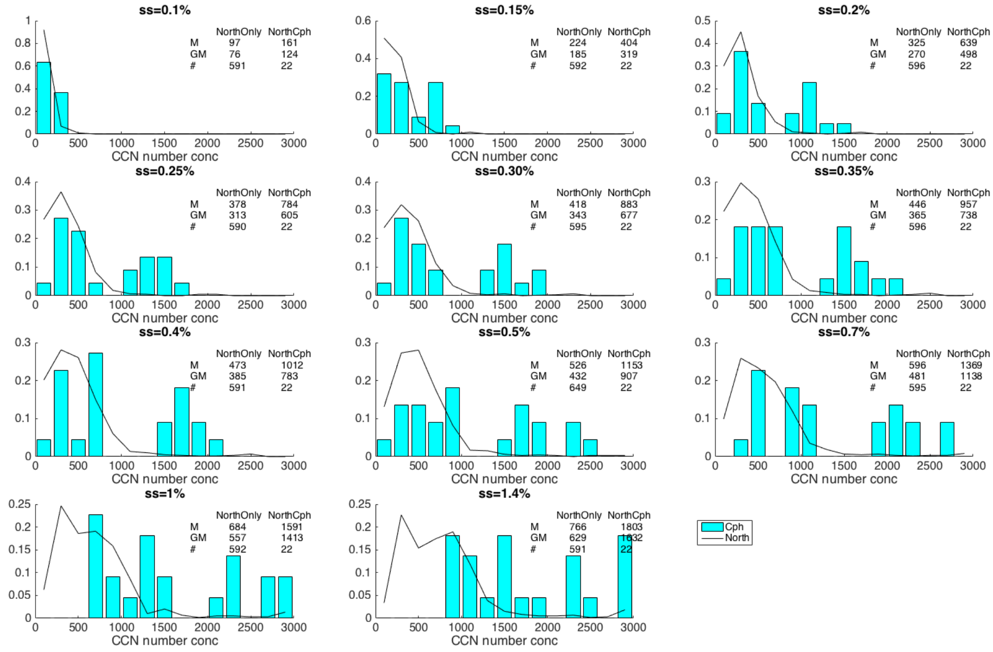

Figure S3. Histograms of cloud condensation nuclei (CCN) concentrations at the rural station with (bars) and without (lines) urban influence for northern air masses. Fraction of data vs concentration (cm^{-3}) at varied supersaturation (ss).

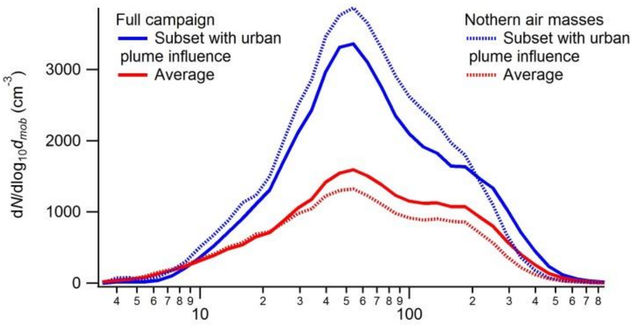

Figure S4. Particle size distributions at the rural station.

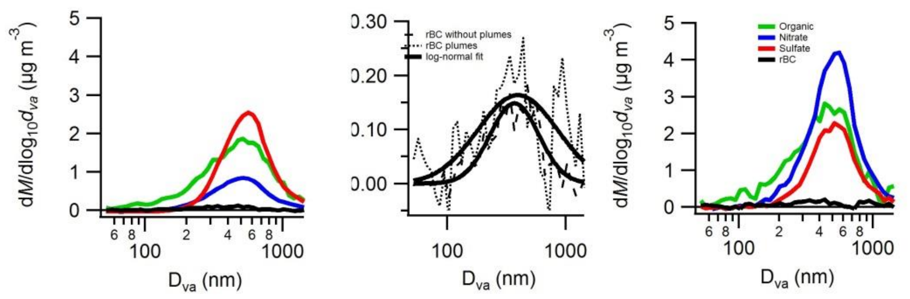

Figure S5. Northen back-trajectories with (right) and without (left) urban plumes. Center: rBC distributions with log-normal fits.

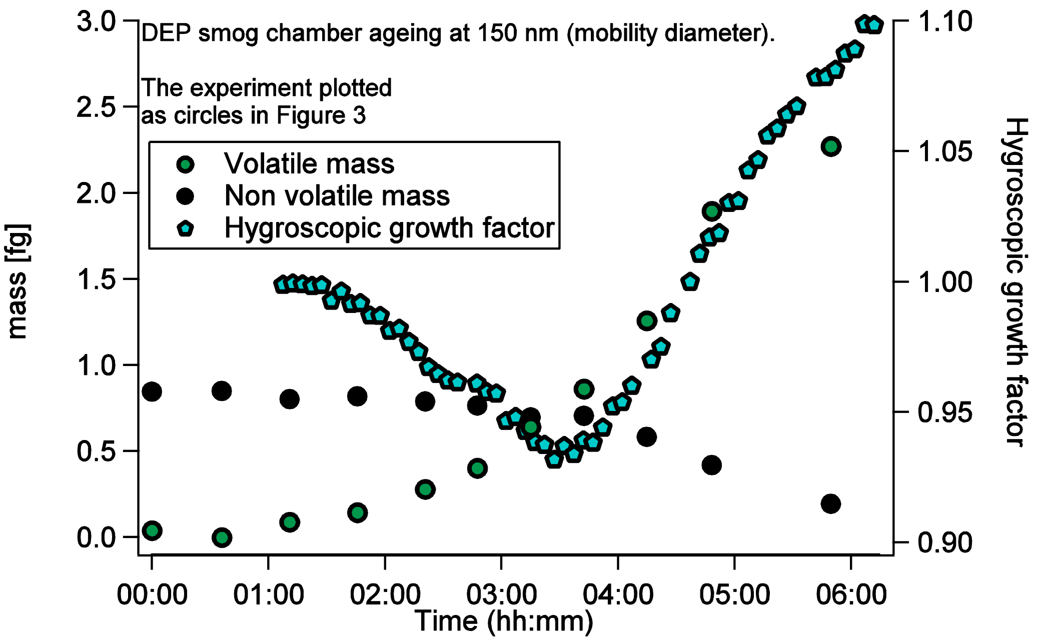

Figure S6. HTDMA and DMA-TD-APM data at Dz 150 nm for one smog chamber DEP experiment (circles in Figure 3). Time 00:00 marks the onset of UV light. A hygroscopic growth factor value of less than one indicates DEP restructuring due to water uptake.

ADCHEM model simulations

Selected Cases:

Case 1. Air mass trajectory passing over Copenhagen near the urban background station H. C. Ørsted Institute (HCØE) on the January 25, 2013 around 11 a.m. and arriving at the rural background station Vavihill (VHL) at 4 p.m., local time. This case represents a typical daytime urban plume event where the secondary nitrate formation in the urban plume is limited by the formation of HNO_3 from the reaction between OH and NO_2. The air mass upwind Copenhagen originates from northern Sweden (Figure S8) and the RH is relatively low (~80 %).

Case 2. Air mass trajectory passing over Copenhagen near the urban background station H. C. Ørsted Institute (HCØE) on January 28, 2013 around 5 a.m. and arriving at the rural background station Vavihill (VHL) at 7 a.m., local time. This case represents a nighttime urban plume event without any photochemical aging. The secondary nitrate formation is caused by the heterogeneous conversion of N_2O_5. The air mass upwind Copenhagen originates from Germany (Figure S8) and the RH is relatively high (~93 %).

Case 3. Air mass trajectory passing over Copenhagen near the urban background station H. C. Ørsted Institute (HCØE) on January 28, 2013 around 9 a.m. and arriving at the rural background station Vavihill (VHL) at 11 a.m., local time. This case represents an urban plume event where the secondary nitrate formation in the urban plume mainly is limited by the formation of HNO_3 from the reaction between OH and NO_2. The air mass upwind Copenhagen originates from Germany (Figure S8) and the RH is relatively high (~94 %).

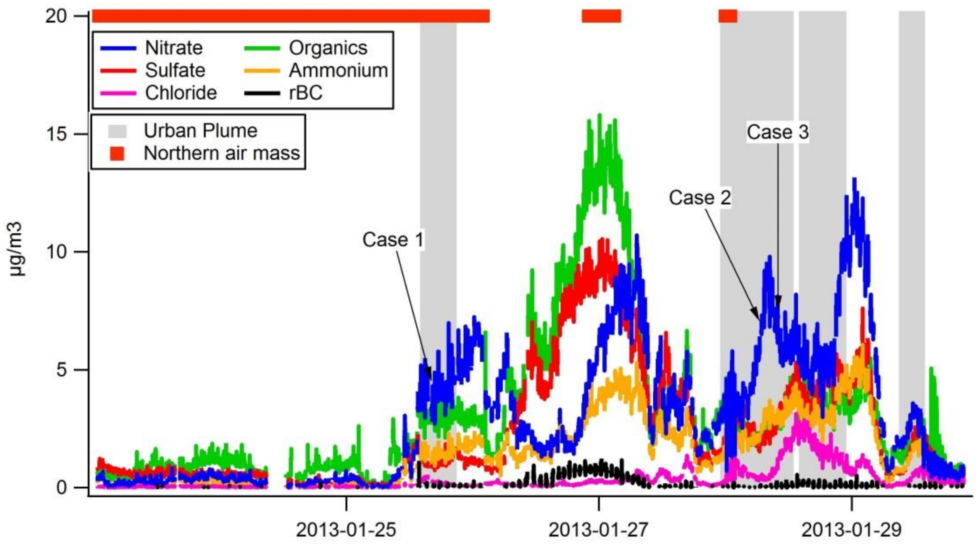

Figure S7. Time series showing the three cases selected for detailed aerosol process modeling. Rural data with urban plume influence shown in grey and northern air masses indicated by the red bar on top.

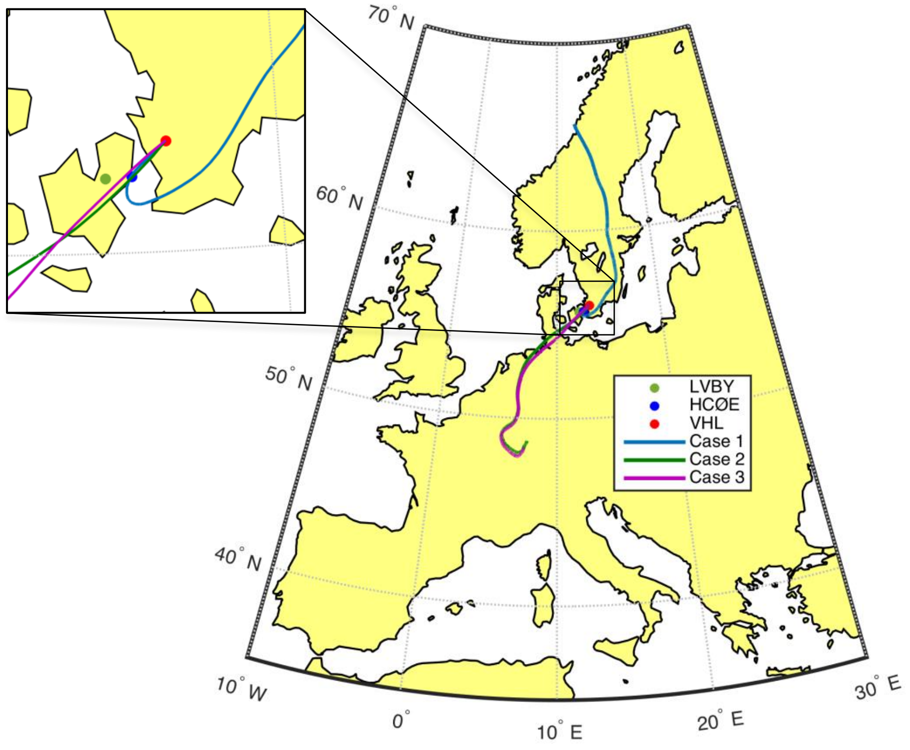

Figure S8. HYSPLIT air mass trajectories for ADCHEM model Cases 1, 2 and 3, and the locations of the three measurement stations: Lille Valby (LVBY, near-city background station), (HCØE, urban background station in Copenhagen) and Vavihill (VHL, rural background station). A detailed description of the three measurement stations can be found in Ketzel et al.[1] The map was created using MatLab version R2014b (https://mathworks.com/products/matlab.html).

Model initialization upwind Copenhagen

To initialize the gas and particle phase concentration and composition we started the model 3 days upwind VHL along the selected air mass trajectories with initially zero particles in each particle size bin. Starting 24 hours upwind Copenhagen, we nudged the modeled particle number size distribution in all vertical model layers within the planetary boundary layer toward the measured particle number size distributions at the near-city background station Lille Valby (LVBY) (Figure S8), which represents the background particle number size distribution ~1-2 hours upwind Copenhagen.

List of volatile organic compounds included in the model description of Cases 1-3:

1-butene, 1-pentene, benzene, acetaldehyde, decane, dodecane, ethyl benzene, ethane, ethene, formaldehyde, 2-butanone, isobutene, m-xylene, o-xylene, p-xylene, butane, nonane, 1,2,3-trimethylbenzene, 1,2,4-trimethylbenzene, 1,3,5-trimethylbenzene, toluene, undecane, propane, propene and phenole.

Model results

Figures S9-S11 show the modeled and measured particle number size distributions at LVBY, HCØE and VHL for Cases 1, 2 and 3. Shown are also the measured and modeled NO_x and O_3 concentrations at HCØE and VHL at the time of the arrival of the air mass trajectories.

Figures S12-S14 show: (a) the modeled total PM2.5 ammonium nitrate (AN) mass, (b) AN mass coating on the externally mixed BC particles originating from Copenhagen, (c) externally mixed BC mass originating from Copenhagen, and (d) the mass growth factor of the externally mixed BC particles (including water) for Cases 1-3.

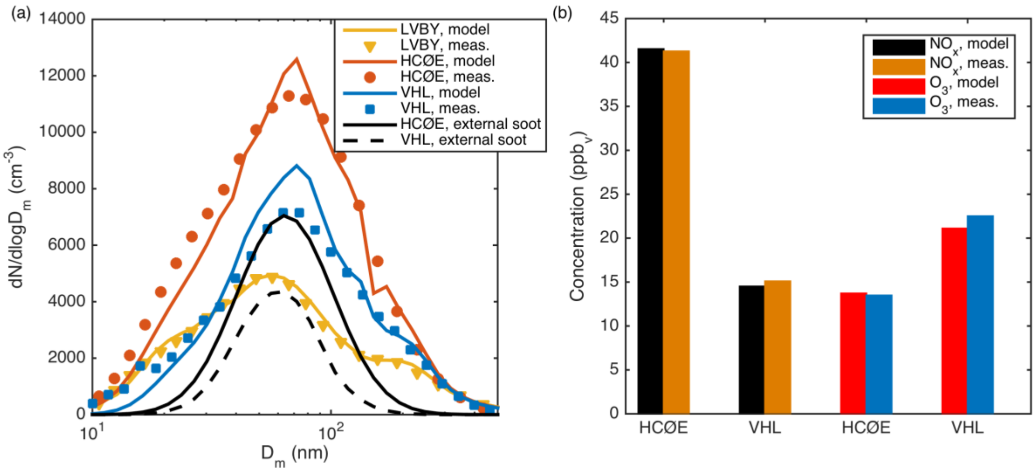

Figure S9. (a) Modled and measured particle number size distributions at LVBY, HCØE and VHL for Case 1, and (b) modled and measured NO_x and O_3 concentrations at HCØÉ and VHL. Shown are also the modled externaly mixed soot particle number size distribution at HCØE and at VHL.

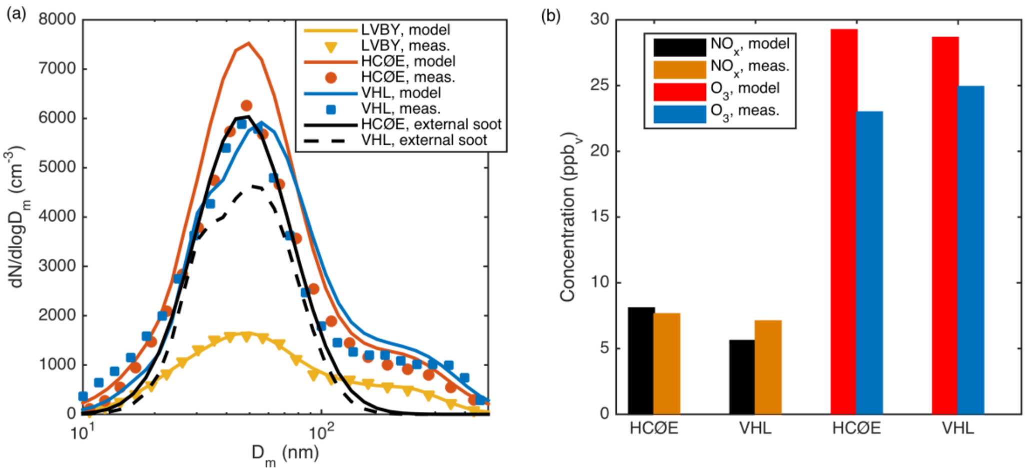

Figure S10. (a) Modled and measured particle number size distributions at LVBY, HCØE and VHL for Case 2, and (b) modled and measured NO_x and O_3 concentrations at HCØÉ and VHL. Shown are also the modled externaly mixed soot particle number size distribution at HCØE and at VHL.

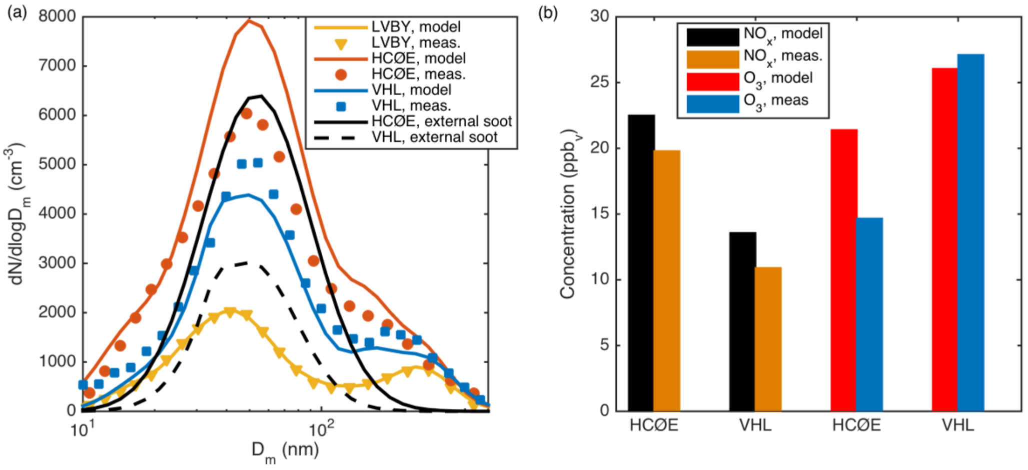

Figure S11. (a) Modeled and measured particle number size distributions at LVBY, HCØE and VHL for Case 3, and (b) modled and measured NO_x and O_3 concentrations at HCØÉ and VHL. Shown are also the modeled externaly mixed soot particle number size distribution at HCØE and at VHL.

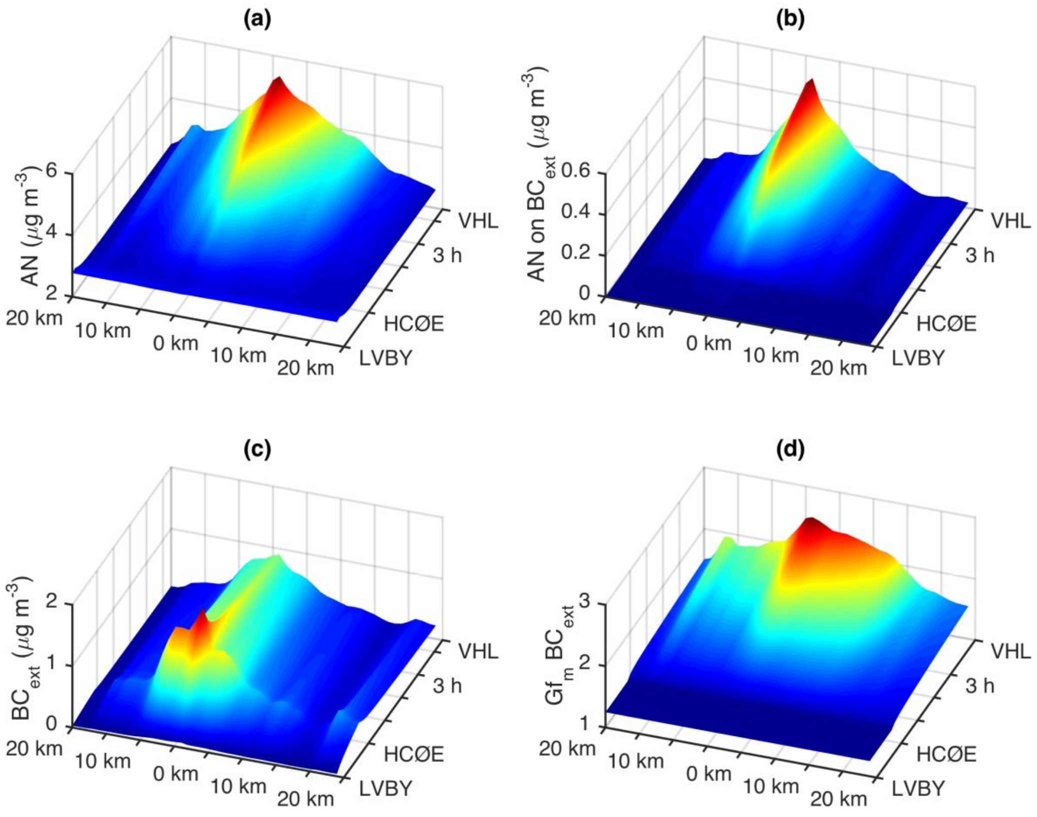

Figure S12. Case 1: Modeled (a) ammonium nitrate (AN) mass, (b) AN mass coating on fresh externaly mixed BC particles from Copenhagen (BC_{ext}), (c) externaly mixed BC particle mass contribution from emission in Copenhagen, and (d) mass growth factor (Gfm) of the BC_{ext} in the urban plume downwind Copenhagen. The Gfm values take into account the growth due to water uptake at the ambient relative humidity, in this case RH=80%.

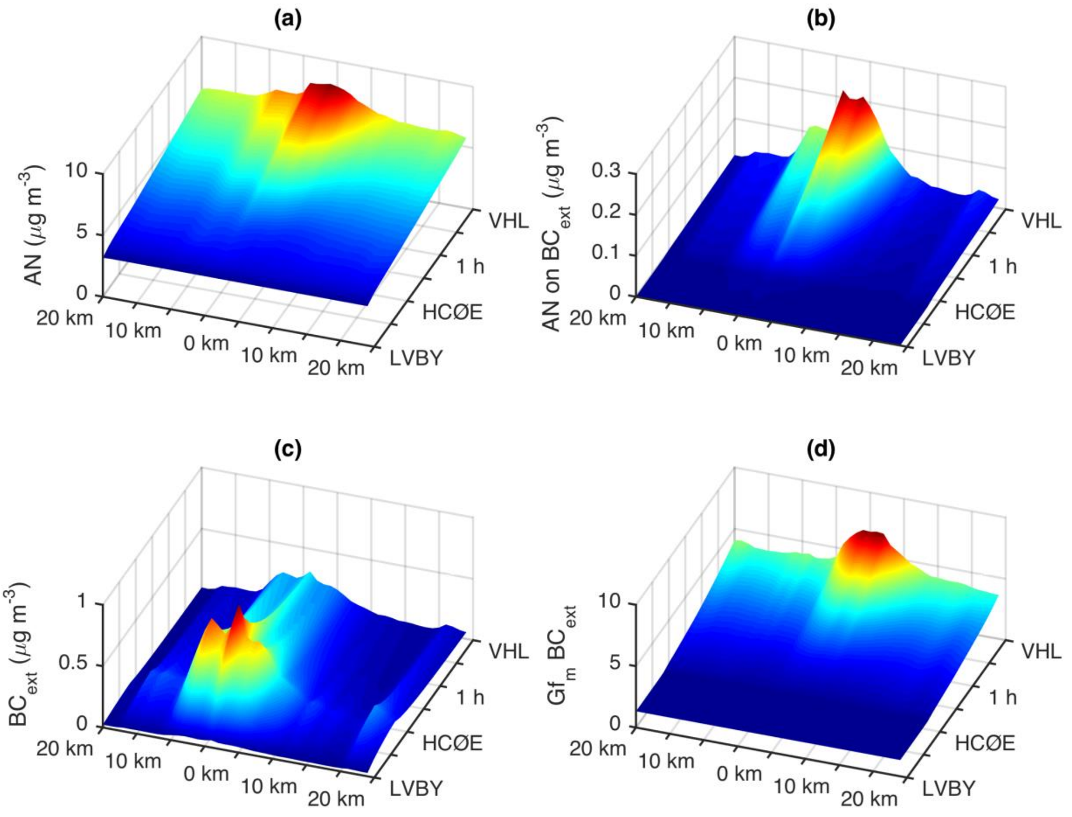

Figure S13. Case 2: Modled (a) ammonium nitrate (AN) mass, (b) AN mass coating on fresh externaly mixed BC particles from Copenhagen (BC_{ext}), (c) externaly mixed BC particle mass contribution from emission in Copenhagen, and (d) mass growth factor (Gfm) of the BC_{ext} in the urban plume downwind Copenhagen. The Gfm values takes into account the growth due to water uptake at the ambient relative humidity, in this case RH=93%.

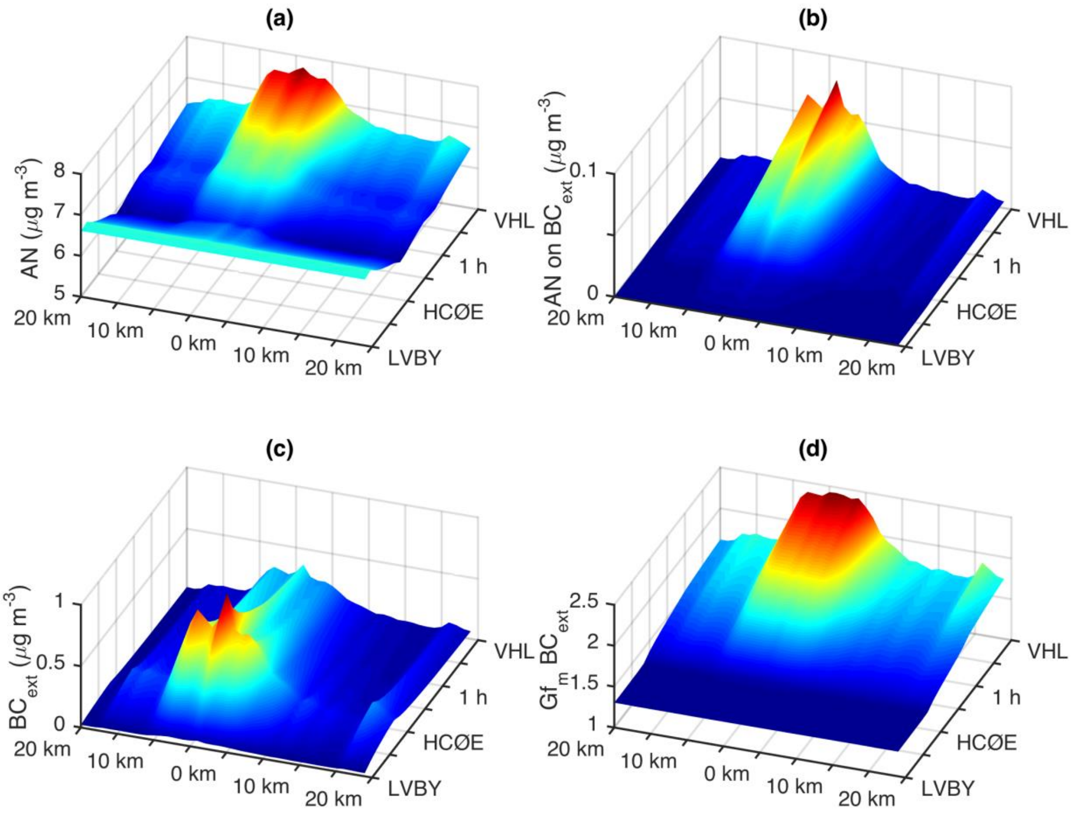

Figure S14. Case 3: Modeled (a) ammonium nitrate (AN) mass, (b) AN mass coating on fresh externaly mixed BC particles from Copenhagen (BC_{ext}), (c) externaly mixed BC particle mass contribution from emission in Copenhagen, and (d) mass growth factor (Gfm) of the BC_{ext} in the urban plume downwind Copenhagen. The Gfm values takes into account the growth due to water uptake at the ambient relative humidity, in this case RH=94%.

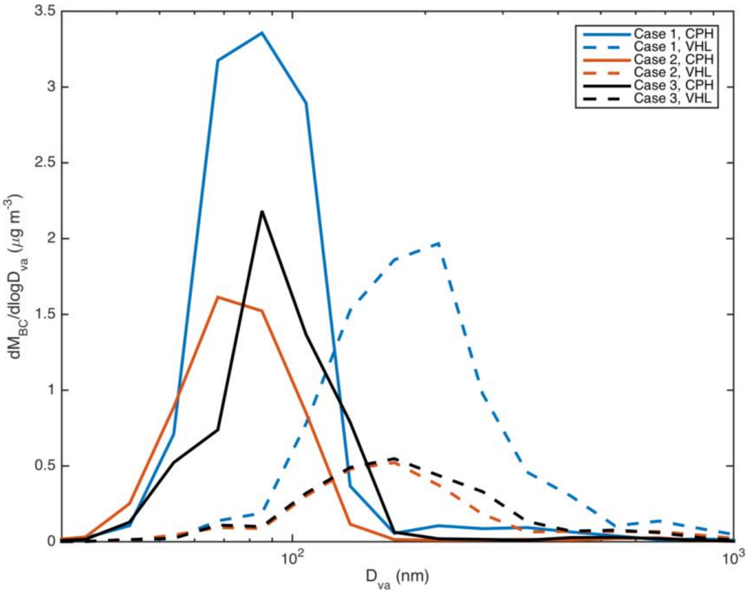

Figure S15. Modeled BC particle mass size distributions in Copenhagen and at Vavihill for Cases 1, 2 and 3.

SP-AMS analysis and calibrations

Black carbon size distributions

The data acquired through aerosol mass spectrometry is two-dimensional: coupled mass-to-charge (m/z), and particle time-of-flight (PToF) are the two quantities measured. PToF is translated into vacuum aerodynamic particle diameter. Half of the sampling time was utilized for non-size resolved acquisition, which has a higher signal-to-noise ratio. As is commonly done, the size resolved data reduction was performed on unit mass resolution spectra, and then corrected based on non-size resolved, high mass-resolution data. The black carbon (BC) size distributions were inferred from m/z 24, which has two contributing ions: C_2^+ and SO^{2+}. Around 0.8% of SO was doubly charged and campaign-wise averages of these contributions were subtracted from the size distributions. C_2^+ was obtained from both black carbon and organic material; the latter yielded mass spectra that consisted of around 0.2% C_2^+, which was subtracted similarly to the sulfate contribution. The approach was verified using mass spectra of non-refractory particulate matter (recorded with the soot module turned off).

SP-AMS calibrations

The SP-AMS was calibrated in the field with 300 nm (mobility) diameter ammonium nitrate and Regal Black particles. Consistent with the findings of Willis et al.[2], the SP-AMS was less sensitive to ambient refractory black carbon (rBC). An effective collection efficiency (CE_{eff}, compared with Regal Black) of 0.32 was applied based on Aethalometer data for the urban campaign. In the rural campaign, a CE_{eff} of 0.67 was found based on thermoptical analysis (OC/EC). Size calibrations were performed with Polystyrene Latex particles. No Regal Black calibration is available for the smog chamber experiments (which predate the calibration procedure introduced by Onasch et al.[3] Therefore the rBC fractions as mesured by SP-AMS were calculated using the default relative ionization efficiencies (1.4 for organic material and 0.2 for rBC) and then scaled to the APM using a factor of 0.9-1.3.

References

1. Ketzel, M. *et al*. Particle size distribution and particle mass measurements at urban,near-city and rural level in the Copenhagen area and Southern Sweden. *Atmos. Chem. Phys.* ***4***, (1), 281-292 (2004).
2. Willis, M. D. *et al*. Collection efficiency of the soot-particle aerosol mass spectrometer (SP-AMS) for internally mixed particulate black carbon. *Atmos. Meas. Tech.* ***7***, (12), 4507-4516 (2014).
3. Onasch, T. B. *et al*. Soot particle aerosol mass spectrometer: development, validation, and initial application. *Aerosol. Sci. Tech.* ***46***, (7), 804-817 (2012).
